# Parents with Intellectual Disability in a Population Context

**DOI:** 10.1007/s40474-015-0042-x

**Published:** 2015-02-26

**Authors:** Gwynnyth Llewellyn, Gabrielle Hindmarsh

**Affiliations:** Centre for Disability Research and Policy, University of Sydney, 75 East Street Lidcombe, Sydney, NSW 2006 Australia

**Keywords:** Parents with intellectual disability, Population studies, Administrative datasets, Vulnerable parent groups

## Abstract

Parenting by people with intellectual disability continues to confront societal sensibilities. On the one hand, parents with intellectual disability engage in the valued social role of raising children; on the other, their parenting attracts (typically negative) attention based on an expectation of their limited capacities to parent. The literature primarily addresses the question of whether or not parents with intellectual disability can be adequate parents or reports on methods for improving their parenting skills. An emerging trend in the literature over the last decade takes a different perspective. Rather than concentrating exclusively on parents with intellectual disability, this perspective focuses on their parenting situation compared to that of other parents more generally. This paper reviews the current state of knowledge about parents and parenting with intellectual disability in this broader population context. The focus of the paper is on the use of larger scale datasets to understand the situation of parents with intellectual disability compared with other parents and to examine the contextual variables that influence their parenting.

## Introduction

The enforced sterilization of people with intellectual disabilities in the early part of the twentieth century is a practice which continues today in some parts of the world [1]. Despite this extreme infringement of human rights, people with intellectual disability have always become parents without regard for the merits or otherwise of their parenting. The first paper in the scientific literature on the topic of parents with intellectual disabilities appeared in the 51st volume of the *American Journal of Mental Deficiency* in 1947 [2]. The author Phyllis Mickelson, reflecting the times, set out to understand the three so-called methods of control of feeble-minded parents: institutionalization, sterilization, and community supervision. She concluded her paper thus “one of the prime purposes of the study was to make sure the use of all methods of control more sure, more knowing and more wise” (p. 653).

Times have moved on from Mickelson’s concern for informing methods to control parents with intellectual disability. However, the conceptualization of their parenting as *parenting in extremis* remains today. This is reflected in the three major themes in the literature which is now in its seventh decade. The first addresses the question of whether people with intellectual disability can provide “good enough” parenting [3]. The second focuses attention on interventions designed to increase parenting skills and, more recently, social supports [4•]. There is also a crosscutting theme evident in Mickelson’s article which remains prominent that relates to concerns about the well-being of the children of parents with intellectual disability including the impact of impoverished home and social environments, child neglect and abuse, and delayed development or disability [5, 6].

The term—parents with intellectual disability—needs further explanation. First, despite this term being frequently used, most studies include only mothers [7]. Several reasons have been advanced to explain the omission of fathers. These include the following: mothers being held responsible for the quality of child care and their child’s developmental progression; antenatal care and post natal support being primarily focused on mothers; and parenting programs primarily addressing maternal skill development. Second, there is the confounding issue of which parents are included in studies found in the literature. As noted in the IASSID (International Association for the Scientific Study of Intellectual Disabilities). Position Statement on Parents and Parenting with Intellectual Disability [5] most parents with intellectual disability have mild or borderline cognitive limitations (IQ 50-75) (http://aaidd.org/intellectual-disability/definition#.VM7lyreKAdU). Parents with intellectual disability come from and continue to experience varying life circumstances. These include the following: (i) people with intellectual disability who were previously institutionalized and now live in the community and have become parents; (ii) people who were diagnosed as intellectually disabled as children and who, living in the community, have been in receipt of specialist services throughout their lives; and (iii) people with intellectual disability who “fly under the radar” of public service surveillance until they become parents at which time their cognitive ability (and hence their parental competence) is questioned. As the literature has matured, the life circumstances of parents with intellectual disability and their children have become relevant variables of interest.

## Research Approaches

An indicator of maturity in the literature is the increasing presence of systematic reviews [8]. Systematic review methods are most useful when there is a substantive literature base. In the literature on parents with intellectual disability, systematic reviews have appeared on parenting skills interventions with the earliest being that by Feldman in 1994 [9]. This review was updated just over a decade later with another seven studies [10]. Within 2 years, the Campbell Collaboration published a systematic review on parent training interventions [11]. The most recent systematic review on parenting skills interventions also addresses interventions to promote social support [4•]. An added benefit of reviews is the identification of gaps in the literature and questions warranting further investigation.

One of the gaps in the literature is the lack of robust data about parents with intellectual disability and their parenting compared to the non-disabled parent population. In other words, most studies have focused on parents with intellectual disability as a special group who are vulnerable with children who are at risk. Very few studies aim to understand their situation in relation to other parents. Developing knowledge about parents with intellectual disability in a broader population context is highly warranted when the merits or otherwise of their parenting is judged on normative grounds [12]. A second and related gap in the literature is the lack of knowledge about the influence of various individual adult, child, family and environmental variables on parents with intellectual disability and their parenting [13, 14]. This is somewhat surprising given the substantive general parent literature on factors associated with the variability in parenting practices. This literature has proven useful in identifying suitable points for intervention [15].

Comparing the situation of parents with intellectual disability and examining contextual variables requires larger samples than can usually be achieved in studies which recruit individuals from clinical services and matched samples. National population surveys and administrative records are two sources of larger scale datasets that offer the opportunity to examine between- and within-group differences. The advantage of national surveys is that a large number of respondents are recruited using sampling strategies that specify representativeness within a defined sampling frame at the population level. This allows for analysis of between-group differences on survey items and also for testing hypotheses of relationships between variables of theoretical interest.

Administrative datasets may be particularly useful for providing detailed information on the situation of people with intellectual disability. These may come from universally available programs such as health care or those determined by eligibility criteria such as social security or housing support. Administrative datasets typically include demographic characteristics such as age, gender, and ethnicity and enable comparisons between populations of people with and without disability. This overcomes one major limitation in studies of people with intellectual disability which primarily draw their adult samples from specialized intellectual disability services. The majority of parents with intellectual disability are those with mild intellectual disability who do not use specialist intellectual disability services, although this varies between countries [16, 17]. Over the last decade, researchers have begun to employ secondary analysis of larger scale datasets to understand parents with intellectual disability in a population context.

The aim of this paper therefore is to discuss the contribution of findings generated by secondary analyses of these larger scale datasets to the current state of knowledge about parents with intellectual disability and their parenting skills. The paper begins with the area in which there is the most advanced understanding of the comparative situation of parents with intellectual disability. This is in relation to maternal health status including pregnancy and birth outcomes and child developmental outcomes. This is followed by a discussion of findings on child protection. The paper concludes with some thoughts about directions for the future of population-based approaches to understanding the situation of parents with intellectual disability and their parenting in a broader population and societal context.

## Health

Health status for parents (primarily mothers) with intellectual disability and their children is one area in which researchers have taken advantage of larger datasets. Most attention has been directed at pregnancy, birth outcomes, and the early post-natal period using hospital/medical registers involving, in some cases, linked datasets. Population surveys have also been utilized to examine children’s health and developmental status in early childhood.

### Pregnancy and Birth Outcomes

In a study by McConnell, Mayes, and Llewellyn [18] utilizing hospital records in Australia, all pregnant women attending their first hospital antenatal clinic visit were screened for intellectual disability using four standard questions in a two-step process and based on a social system definition as follows: “Have you ever been in a class or school for students with learning difficulties?” and “Do you receive a pension or benefit for a disability?”. If affirmative, two additional questions confirmed school placement due to generalized learning difficulty (not dyslexia) and that the disability pension was received for intellectual disability rather than other types of disability. Post-partum, pregnancy and birth outcomes were extracted from their medical records. A cohort of 54 (6.5 %) women was identified with intellectual disability from the population of 834 women (839 newborns). The rate of preeclampsia (i.e., high blood pressure, fluid retention, and protein in the urine) observed was significantly higher in women with intellectual disability compared to women without intellectual disability (22.2 vs 9.1 %; odds ratio [OR] = 2.85). There was little difference between the two groups of women on three other conditions of concern in pregnancy: diabetes mellitus, gestational diabetes, and essential hypertension. There were no significant differences in time of first antenatal clinic visits or the total number of visits. There were however significant birth outcome differences. Women with intellectual disability had higher rates of low birth weight (<2,500 g) babies (14.5 vs 5.2 %, OR = 3.08), and their babies were more likely to be admitted to special care (23.6 vs 11.0 %, OR = 2.51).

Goldacre, Gray, and Goldacre [19] utilized an archived dataset, the *Oxford Record Linkage Study* (OLRS), which links records “on hospital admissions, maternity and delivery records, and birth and death registrations in a defined geographical area of South East England” (p. 2). Between 1970 and 1989, there were 217 (0.09 %) births to mothers with intellectual disability out of a total 245,007 births. Overall, mothers with intellectual disability were more likely to be younger, of low social class, and higher parity. They were also much more likely than other mothers to be unmarried (42 vs 9 %) and to have smoked in pregnancy (55 vs 23 %). There were, however, few significant differences in terms of birth outcomes. Women with intellectual disability were more likely to have babies with fairly low birth weights (2,000–2,499 g and 2,500–2,990 g) although very low birth weights (<2000 g) were similar across both groups at 1.9 and 1.8 %. Due to the historical nature of the data, the authors adjusted for social class and year of birth and found that “significant associations remained between intellectual disability and young maternal age, unmarried motherhood, maternal smoking, low birth weight and not breastfeeding” (p. 5).

In Sweden, Höglund and colleagues investigated pregnancy and birth outcomes [20•] and perinatal health and death in children born to mothers with intellectual disability [21]. Their papers report on secondary analysis of data from two linked population health registers over the period 1999–2007: the *National Patient Register* and the *Medical Birth Register*. There were 326 (0.096 %) first time, singleton mothers with intellectual disability and no psychiatric diagnoses in the dataset. This cohort was compared with all first time, singleton mothers without intellectual disability or any other psychiatric diagnoses (*n* = 340,624). A higher proportion of women with intellectual disability were teenagers, obese and single, and these women smoked more than women without intellectual disability. In the first paper [20•], they report significant differences between the groups on preterm birth (12.2 vs 6.1 %, OR = 1.68), a caesarian section (24.5 vs 17.7 %, OR = 1.55), non-use of nitrous oxide (i.e., laughing gas) (59.5 vs 75.8 %, OR = 1.89), and discharge from hospital to a place other than home (6.5 vs 2.4 %, OR = 2.24). In the second paper [21] which focuses on the neonatal period, they found that stillbirth was almost four times more prevalent among babies born to women with intellectual disability than among those born to mothers without intellectual disability. Perinatal death similarly was more than four times more common among babies born to mothers with intellectual disability (1.8 %) than among babies born to mothers without intellectual disability (0.4 %). In multivariate analyses, intellectual disability was associated with preterm birth, cesarean section, non-use of nitrous oxide, stillbirth, perinatal death, Apgar score <7 (at 5 min), and discharge to a place other than home.

### Health Status, Health Behaviors, and Child Outcomes

The focus on pregnancy, birth outcomes, and the neonatal period has broadened recently to include the investigation of health status (primarily of mothers) and child outcomes. The increasing availability of large scale representative national population studies allows for examination of the intersection of maternal, child, family, and environmental factors. When these studies are longitudinal, there is an added advantage in identifying factors or combinations of factors that influence mother’s, father’s, and children’s trajectories over time. In the UK, this has become possible with the introduction of nationally representative longitudinal studies in this case, the *Millennium Cohort Study* (MCS) designed to follow, throughout their lifetime, a nationally representative group of children born in the UK between September 2000 and August 2001.

Hindmarsh, Llewellyn, and Emerson [22•] utilized secondary analysis of the first wave of the MCS to examine the health and social context of mothers with intellectual disability and their 9-month-old infants. The data collected at wave 1 included circumstances during pregnancy, birth, and the first 9 months of life as well as the social and economic background of the family. Of the 18,189 mothers at wave 1 (9 months of age), 74 (0.4 %) were identified as having an intellectual disability, defined for this purpose as low educational attainment and poor numeracy and literacy skills. These mothers were significantly younger at the birth of their first child, less likely to be married, or have the natural father residing with them, more likely to be the only parent/carer in the house, financially worse off in terms of employment, housing, being in receipt of government benefits and lacking assets.

Details about birth outcomes were provided by respondent recall. Assessment of maternal mental and physical health status involved a combination of standardized measures, self-report, and forced choice items. Where possible similar birth outcome measures found in the studies reviewed above were examined including low birth weight, preterm birth, problems at birth/first week of life, admitted to Special Care Nursery, and days old at discharge. Birth outcomes for the infants of mothers with intellectual disability compared to infants of mothers without intellectual disability were not markedly different with one exception: infants of mothers with intellectual disability were more likely to be older at discharge.

Mothers with intellectual disability were more likely to self-report their health as fair/poor, have a longstanding illness/disability, and smoke (at wave 1). These mothers were significantly less likely to consume alcohol and there was no difference in body mass index between mothers with and without intellectual disability. While mothers with intellectual disability reported higher rates of mental health problems (self-report on feeling sad, diagnosis of depression by a doctor, and a standardized instrument measuring depression), they were not significantly different from mothers without intellectual disability. Similarly, on the psychological well-being indicators, there were no differences between the two groups, with the exception that mothers with intellectual disability were twice as likely to strongly agree/agree with the statement “I am inclined to feel that I am a failure” and were more likely to report their satisfaction with life lower/poor than mothers without intellectual disability. Ongoing analyses of the MCS are addressing the influence of child characteristics, parental health and family context, and environmental factors on children’s social and emotional well-being over time [23].

Recent papers from Emerson and Brigham also address the health status and health behaviors of parents with intellectual disability [24], and the developmental health of their children [25•]. These authors conducted a secondary analysis of a confidentialized needs analysis dataset collected from three Primary Care Trusts in England, covering a population of 1.25 million people. Data on health, social, and lifestyle situation of the family and details of illnesses and disabilities were collected by health visitors using standardized definitions and a common survey form. These data were collected at household level (*n* = 46,023); parent gender and age of child/children were not reported (although all children were under 5 years). Among the 5,256 (11.4 %) single parent households, 3.2 % were identified as having a parent with intellectual disability. Among two parent households, 1.0 % were identified as having one or more parents with intellectual disability.

Analyses were conducted separately for single and two parent households with and without a parent (or parents) with intellectual disability. To examine parental health status and health behaviors, analyses addressed alcohol abuse, drug abuse and smoking health behaviors, mental health status, and 13 environmental adversities [24]. Households with a parent with intellectual disability were significantly more likely to be exposed to adversities such as low income, unemployment, unstable housing, and social isolation. Across both single and two-parent-headed households, those with a parent with intellectual disability had significantly poorer mental health. Analyses in which between group differences in socio-economic position (e.g., housing, income), social isolation and other adversities (e.g., violence within the family) were controlled eliminated the increased risk of poorer health for single parent households headed by a parent with intellectual disability.

In their second paper utilizing the same dataset, Emerson and Brigham [25•] examined the four child developmental outcomes recorded by the health visitors: developmental delay, speech and language problems, behavior problems, and frequent accidents and injuries. Significantly higher rates of poorer child outcomes were found for households including a parent with intellectual disability across all four measures. Significantly higher rates of exposure to environmental adversity were found for households with a parent with intellectual disability on 17 of the 18 indicators of environmental adversity. Risk on the four measures of child developmental health was significantly reduced by adjusting for between group differences on socioeconomic position and on environmental adversity.

## Child Protection

Secondary analysis of large datasets has been employed most frequently to examine prevalence and outcomes for parents with intellectual disability and their children in statutory child welfare proceedings. This focus was driven initially by observations that (i) in clinical samples of mothers with intellectual disability, a high proportion of mothers had their children removed (e.g., [26]) and (ii) of mothers with children in care, a high proportion had a disability (e.g., [27]). The findings from these earlier studies suggested that on average 40 % of the children of parents with intellectual disability were no longer living with their biological parents [28].

Child protection authorities are required to maintain relatively fulsome records on family, case, and court process characteristics. Analysis of court file records permits between- and within-group comparisons on specified parent groups. Llewellyn, McConnell, and Ferronato [29] reviewed court files of the 285 cases involving 469 children finalized over a 9-month period in 1998–1999 at two Children’s Courts in NSW Australia. Nearly one third of the 285 cases featured parental disability; 9 % of all 285 cases were parents with intellectual disability. The highest prevalence was parental psychiatric disability (e.g., psychotic, mood, anxiety and personality disorders) at 22 %. There was a relationship between parental disability (i.e., intellectual, psychiatric, physical, and sensory) and the outcome of the court proceedings, with the more extreme outcome of the child being removed from their parents and placed in out-of-home care occurring more frequently for children of parents with intellectual disability than for other parent groups.

Using a similar approach, Booth, Booth, and McConnell [30] reported a documentary review of 437 care applications concerning 828 children from four courts in the north of England in the year 2000. They found more than one in six of care applications in which there was at least one parent with intellectual disabilities. This study also found a relationship between parental disabilities type (i.e., intellectual, physical, and sensory disabilities, mental illness, and drug/alcohol issues) and court outcome. The children of parents with intellectual disability were significantly more likely to be put up for adoption (41.7 %) compared to the children of parents with no intellectual disability (28.9 %). Further, there was a significant association between disability type and placement outcome such that the children of parents with intellectual disability were significantly more likely to be placed in out-of-home care than the children of parents with the other disabilities.

In a subsequent paper, the same authors presented a cross country comparison study based on the findings from the Australian and English court studies [31]. The country comparisons demonstrated that parents with intellectual disability were proportionally over-represented in child protection proceedings in both countries, although the over representation was greater in England than in Australia. The authors point to the difficulty in quantifying this over-representation as a proportion of the total population of parents with intellectual disability in either country. This is due to the lack of reliable prevalence data on parents with intellectual disability although the estimated number of parents with intellectual disability is usually reported at around 1 % of the total population [32]. Differential outcomes were observed for the children of parents with intellectual disability in the Australian sample compared to the English sample with those in England being more likely to be permanently placed out of home. These differences could reasonably be explained by the care and protection policies in each country, the English favoring earlier adoption, and the Australian favoring family re-unification.

Court file data as utilized in these Australian and English studies offer larger scale samples and the opportunity to investigate between group differences. Review of court records is however highly resource intensive; thus, only a small number of geographical locations were selected in both the Australian and British studies. Resource constraints would most likely prevent this method being used at a state or national level given for example the annual number of court orders in Australia now exceeds 50,000 [33]. However, the (potentially) non-representative sampling of court records from particular court locations limits the generalizability of the findings.

In Canada, the presence of a large national representative sample of child maltreatment investigations overcomes this limitation and offers additional opportunities to investigate differences between parents with intellectual disability and other parent groups on a number of dimensions. The dataset is the *Canadian Incidence Study of Child Abuse and Neglect* (CIS) core-data which are derived from a multi-stage stratified cluster sample of child maltreatment investigations from each Canadian province and territory, except Quebec [34]. Data are collected by in-depth standard survey completed by child welfare investigators from administrative data and their knowledge of the case. This dataset offers the opportunity to examine differences in prevalence and predictors of child maltreatment investigation substantiation, case dispositions, applications to the child welfare court, and the influence of mediating variables on investigation outcomes. It also offers the opportunity to test hypotheses derived from theoretical models of parenting.

In their first study using the CIS-2003, McConnell, Feldman, Aunos, and Prasad [35] investigated the prevalence of parental cognitive impairment (parents with intellectual disability and parents with borderline intellectual functioning) in the 11,562 cases opened for child maltreatment investigation as well as the outcomes of these cases. The prevalence of parental cognitive impairment was 10.1 %. In the cases involving parents with cognitive impairment, neglect was by far the most common reported child protection concern. There was some empirical support for the hypothesis that psychosocial risk (e.g., poverty, mental health issues, and low social support) partially mediated the relationship between parental cognitive impairment and child maltreatment investigation outcomes. As the authors note, there are limitations utilizing a dataset derived from professional reports (in the survey) compared to, for example, data collected on standard measures. The influence of professionals’ perceptions resulting in systematic bias cannot be ruled out. It could be the case, for example, that low social support and/or mental health issues influenced professionals to categorize parents as cognitively impaired which then led to more “extreme” child maltreatment investigation outcomes rather than psychosocial risk mediating the relationship between parental cognitive impairment and investigation outcomes.

In a second study, the same authors [36] investigated in-depth 1,243 child investigations within the same dataset (CIS-2003) in which parental cognitive impairment was noted. The sample size permitted examination of child, case and parent and household “risk factors” for the four investigation outcomes of substantiation, cases kept open with and without referral to other services, and court application. There are several findings from this study that have implications for practitioners. The first is that perceived parent noncooperation was a strong predictor of court application. Second, there was relatively little use by professionals of alternative dispute resolution as an appropriate first course of action. This seems unusual given that this course of action is the preferred approach for other parents entering the child protection system. The third is that parent social isolation/few social supports were strong predictors of cases being kept open even in the event of lack of substantiation of maltreatment. A fourth finding is that neglect was by far the most common form of child maltreatment confirming the findings from the court records in Australia and the UK described above [29, 30].

In a third study, Feldman, McConnell, and Aunos [37] utilized three measures of child functioning (emotional/behavioral, learning/development, and disabilities/health) within the CIS-2003 dataset. Based on Feldman’s interactional model of parenting [13], they created four sets of predictive factors: contextual variables, parent health and well-being, type of alleged maltreatment, and child characteristics. Two thirds of the sample of children did not have a learning/development problem. Forty-two percent of the sample had no identified child functioning issue. As the authors observe, this finding is in stark contrast to the persistent concerns by care and protection practitioners about the developmental outcomes of children of parents with intellectual disability [38, 39]. Low parental social support and mental health issues predicted child functioning with parental mental health mediating the relationship between social support and child outcome.

The concern by practitioners about the social isolation of parents with intellectual disability and poor or inappropriate support has led to an increase in interventions to increase (particularly) mothers’ social supports and community participation [40, 41]. The findings reported here from the health and child protection areas confirm the importance of social support for the well-being of children of parents with intellectual disability. Collings and Llewellyn [42•] reported a similar finding in their review of outcomes for children of parents with intellectual disability. In an empirical study, Wade, Llewellyn, and Matthews [10] demonstrated that access to social support predicts parenting practices, which in turn predicts child well-being with the context influencing child well-being through the mediator of parenting practices. Given the increasingly robust evidence for social support as a critical contextual influence on child well-being, Wade et al. conclude with a call for “empirical research that examines factors (e.g., family history, socio-economic circumstances, geographic location) that influence the social support available to parents” (p. 431). In the meantime, the evidence to date suggests that practitioners would be wise to expand parenting skills training programs to include interventions that have shown promising results in increasing social supports for parents with intellectual disability [40, 41, 42•, 43, 44].

## Conclusion

From the population-based studies to date on health status, the standout findings are the following: (i) that from the beginning, mothers with intellectual disability are more likely to experience several risk factors of pregnancy including younger maternal age, single parenthood, low birth weight newborns, poorer mental health, and lower socioeconomic position and (ii) that in the early years, parents (not disaggregated by sex) with intellectual disability also experience poorer mental health, socioeconomic circumstances and environmental adversities.

From the secondary analysis of administrative data on child protection, the standout findings are the following: (i) that parents with intellectual disability are significantly over-represented in child protection proceedings with a higher likelihood of their children being placed into care and (ii) that low parental social support and mental health issues directly influence child developmental outcomes.

The findings on child developmental outcomes to date from population-based studies are less clear cut. The data in the Canadian child protection dataset analyzed by Feldman et al. [37] show less than half (42 %) of children had no identified functioning issue. In contrast, the British data sourced by Emerson and Brigham [25•] show significantly higher rates of poorer outcomes on four measures of child developmental health for children of parents with intellectual disability compared to their peers. However, the risk was significantly reduced by adjusting for between group difference on socioeconomic position and on environmental adversity.

For practitioners, the implications are clear about areas of focus in working with parents with intellectual disability. The first is the need to address parental social skills, relationships and networks to reduce social isolation and increase social support. A flow on effect could be anticipated as there is good evidence from social support interventions more broadly that social participation is associated with better mental health in the general parent population. A specific focus on the mental health needs of mothers would also be beneficial. This is particularly so as Mayes and Llewellyn [45] have pointed out in relation to the presence of child protection in the lives of many parents with intellectual disability and their grieving following child removal. A further benefit in attending to maternal mental health is the “protective” feature of better parental mental health and good social support for the well-being of children.

The advances in computing power brought directly to the desk (or notebook) have enabled secondary analysis of larger scale datasets previously beyond the reach of individual researchers. Standard disability questions or a disability module in national population health, social, and labor surveys would further increase the capacity to disaggregate disability data by demographic characteristics such as age, sex, race, and socioeconomic status. Critically, disability questions in longitudinal and life course surveys offer the opportunity to examine patterns and trends for people with disabilities compared to their non-disabled peers as well as information about subgroups of people over time. In a similar vein, having a standard disability “flag” that identifies records of people with a disability in administrative data collections (such as housing, education, employment, child protection) offers the opportunity to examine demographic characteristics of recipients (service users), types of services, and inputs and outputs for people with disabilities compared with their non-disabled counterparts. These initiatives are progressing apace with researchers in the intellectual disability field capitalizing on the opportunities offered.

The major advantages of these approaches are access to large representative samples in a population context; the capacity to use robust analysis methods to examine between- and within-group differences and to investigate disadvantage and inequity; the opportunity to test hypotheses on variables of interest and their interactions; in the case of registers and administrative datasets, the potential capacity to link to other registers and administrative data across the life domains; the capacity to answer questions bringing data together from several sources and to do so with potentially vulnerable or “over-researched” populations without additional data collection; and to do all the above at substantially reduced personnel and financial cost compared to studies requiring recruitment of individuals according to specified criteria.

An additional advantage accrues where the surveys are longitudinal and intended to address the life course. This moves understanding beyond cross-sectional comparisons and between-group differences on variables at a “point in time” to examining factors that influence individual (child or parent) and family trajectories over time. In the UK, this is possible with the introduction of nationally representative longitudinal studies such as the *Millennium Cohort Study*. Datasets such as this offer the opportunity to explore inequities and disadvantage not only within parent and child generations but also across generations over time. This seems particularly pertinent to women with disability who become mothers given their increased likelihood of experiencing well-documented risk factors in pregnancy.

Research on intellectual disability in a population context is anticipated to continue to increase in line with expanding international interest in the life circumstances of people with disabilities compared to their non-disabled peers. As this occurs, identifying appropriate points for intervention and interventions which effectively target inequities and disadvantage can only be in the best interests of parents, their children, and society more broadly.
